# High lncSNHG15 expression may predict poor cancer prognosis: a meta-analysis based on the PRISMA and the bio-informatics analysis

**DOI:** 10.1042/BSR20194468

**Published:** 2020-07-17

**Authors:** Shi Xu Fang, Cheng Chen, Qiang Guo, Xi Xian Ke, Hong Ling Lu, Gang Xu

**Affiliations:** 1 Department of Thoracic Surgery, The Affiliated Hospital of Zunyi Medical University, 149 Dalian Road, Zunyi, Guizhou 563000, China; 2 Department of Biochemistry, Zunyi Medical University, No. 6 Xuefu West road, Xinpu New Area, Zunyi, Guizhou 563099, China

**Keywords:** cancer, LncRNA, meta-analysis, prognosis, SNHG15

## Abstract

Background: *SNHG15* has been reported to be aberrantly expressed in various tumor tissues and could serve as a promising prognostic cancer biomarker. Previous studies on *SNHG15* yielded inconsistent results with insufficient sampling. Here, a meta-analysis was conducted to investigate the prognostic value of *SNHG15* in multiple cancers. Methods: Relevant studies were retrieved from six electronic databases including PubMed, Cochrane Library, Google Scholar, Embase, Web of Science and China National Knowledge Infrastructure (CNKI). Fifteen publications comprising 1318 patients were included. The publication bias was identified by the Begg’s Test, and the sensitivity analysis was also performed. Results: The results demonstrated a positive correlation between high expression level of lncSNHG15 and short overall survival (hazard ratio (HR) = 2.07, 95% confidence interval (CI), 1.48–2.88; *P*<0.0001) and disease-free survival (DFS) (HR = 2.32, 95% CI, 1.53–3.53; *P*<0.0001). The analysis based on different cancer types showed that SNHG15 had the most prominent prognostic potential in Glioma (HR = 3.81; 95% CI, 0.84–42.69; *P*=0.28). Moreover, the high expression level of lnc*SNHG15* indicated advanced TNM stage (OR = 2.52; 95% CI, 1.33–4.76; *P*=0.00001), lymph node metastasis (OR = 2.41, 95% CI, 0.99–4.81; *P*=0.05), bigger tumor size (OR = 2.06; 95% CI, 1.03–4.13; *P*=0.04) and poor histological grade (OR = 2.62, 95% CI, 1.90–3.59; *P*<0.00001), yet no association with distant metastasis (OR = 1.64, 95% CI, 0.40–6.74; *P*=0.49), age (OR = 0.98, 95% CI, 0.78–1.22; *P*=0.84) and gender (OR = 0.9, 95% CI, 0.71–1.14; *P*=0.3838) was found. Its conclusions further confirmed by exploring TCGA databases. Conclusion: It revealed that lnc*SNHG15* might be a promising prognostic biomarker of multiple cancer types, especially in Glioma.

## Introduction

The cancers have attracted the attention of researchers due to their insidious onset, difficulty to cure and heavy financial burden of treatment [1,2]. Annual cancer-related morbidity and mortality rates continued to increase in the United States in 2019 [3]. The same severe phenomenon could be seen in China; the number of cases and deaths per 100,000 people per year were 733 and 610 respectively in China in 2015 [4]. Although an ever increasing number of researchers are involved in cancer researches and many new treatments and drugs have emerged in recent years, many patients have been still diagnosed already in middle and late stages of cancer due to atypical early symptoms, thus missing the best time for treatment [5]. Therefore, cancer prognosis is still not ideal [6]. Hence, researchers have recently turned their attentions to identify the molecular mechanisms of cancers to find more promising therapeutic targets and reliable prognostic biomarkers.

In recent years, long non-coding RNA has become the focus of many researchers. As we all known that only 2–3% of all DNA transcription products (RNA) in human genome have protein coding ability [7], whereas up to 95% of RNA molecules do not code a protein (non-coding RNA) [8]. Non-coding RNA longer than 200 nucleotides is termed as long non-coding RNA (lncRNA). LncRNA does not have a single and pre-defined biological function [9]. Decades of lncRNA researches showed the involvement of lncRNA in the activation and silencing of genes, nuclear transfer and chromatin modification [10]. More evidences have confirmed that the abnormal expression of lncRNA could be found in various tumor tissues as well. Furthermore, the aberrant expression of lncRNA was shown to be associated with the onset and development of cancers [11]. For example, *lncUCA1* was highly expressed in hepatocellular carcinoma tissues and closely associated with larger tumor sizes, high risks of vascular invasion, advanced TNM stages and bad survival outcomes [12]. *lncMALAT1* was highly expressed in lung cancer tissues and closely related to the proliferation and invasion of lung cancer cell [13]. *lncDANCR* was highly expressed in hepatocellular carcinoma tissues and significantly correlated with cancer progression [14].

*SNHG15* is 837bp in length and located on human chromosome 7p13 (https://www.ncbi.nlm.nih.gov/nuccore/NR_003697.1), a highly conserved long non-coding RNA [15], and it was initially found to act as an oncogene with up-regulated expression in hepatocellular carcinoma cells [16]. Recent studies reported an aberrant expression of *SNHG15* in various malignant tumors including liver cancer [17], breast cancer [18], gastric cancer [19], lung cancer [20–23], glioma and colorectal cancer [24,25]. The abnormal expression of *SNHG15* promoted the proliferation, invasion and epithelial–mesenchymal transition (EMT) of tumor cells as well [26]. In addition, an abnormal expression of SNHG15 was reported with a marked relationship with cancer prognosis [27]. Therefore, *SNHG15* is closely related to various biological behaviors of tumor cells and may serve as a potential therapeutic target and an indicator for cancer prognosis. However, previous studies on this lncRNA were limited in sample sizes and yielded inconsistent results. Here, a meta-analysis was performed to assess the prognostic value of SNHG15 in cancers.

## Materials and methods

### Retrieval of studies

This research was undertaken in accordance with the statement of the Preferred Reporting Items for Systematic Reviews and Meta-Analyses (PRISMA) [28]. In order to include all related publications, several electronic databases were searched including PubMed, Cochrane Library, Google Scholar, Web of Science, EMBASE and China National Knowledge Infrastructure (CNKI, including studies deposited up until June 1, 2019). The keywords were (“small RNA host gene 15” or “SNHG15” or “long non-coding RNA SNHG15” or “lncSNHG15”) and (“cancer” OR “tumor” or “carcinoma” or “malignancy”) and (“prognosis” or “prognostic” or “outcome”). The references of primary literature were also searched.

### Inclusion and exclusion criteria

Studies were selected by two independent researchers. Inclusion criteria were as follows: (1) investigation of the correlation between the expression of *SNHG15* and survival outcome as well as clinical prognosis of patients with cancers; (2) classification of patients into high and low expression groups in accordance with primary literatures; (3) detection of expression level of *SNHG15* by validated techniques; (4) sufficient data to calculate odds ratio (OR) or hazard ratio (HR); and (5) studies written in English or Chinese. The exclusion criteria were: (1) no investigation of the relationship between the expression level of *SNHG15* and cancer prognosis apart from a mere exploration of the involved molecular biological mechanisms; (2) reviews and meta-analyses, letters, animal studies and conference proceedings; (3) insufficient data for prognostic analysis; (4) duplicate studies.

### Quality assessment of selected studies

**Figure 1:**
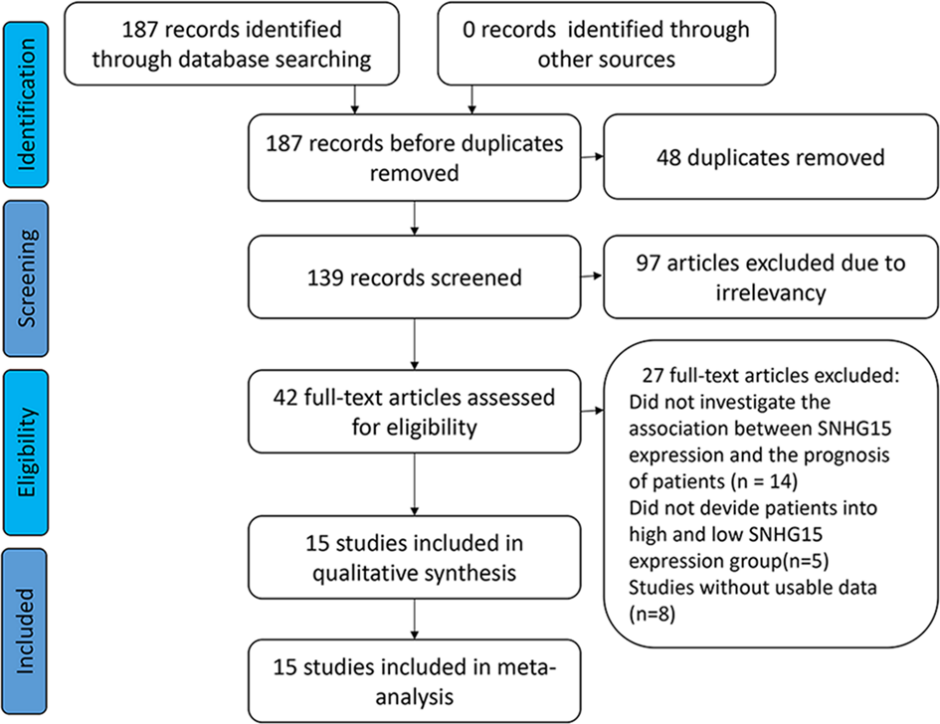
Flow diagram of the eligible studies

Two researchers (X.G. and L.H.L.) independently scored the selected studies using the Newcastle–Ottawa Quality Assessment Scale (NOS) to assess the quality and suitability of the selected studies for inclusion in this meta-analysis (http://www.ohri.ca/programs/clinical_epidemiology/oxford.asp). The NOS items of selection of cohorts, comparability among included studies and outcome were included [29]. The study selection flow diagram is shown in Figure 1.

### Data extraction

Two researches (C.C. and F.S.X.) independently extracted the following items from selected studies: (1) the first author(s); (2) year of publication; (3) area of patients; (4) types of cancers; (5) detection methods; (6) number of patients; (7) cut-off values and follow-up time; (8) clinical pathological data including lymph node metastasis (LNM), TNM stage, distant metastasis (DM), tumor size, gender and age; (9) survival outcome including overall survival (OS), disease-free survival (DFS) and progression-free survival (PFS). In cases where both univariate and multivariate analysis results were provided, results from multivariate analysis were extracted. If only the Kaplan–Meier curve was presented, the Engauge Digitizer V 4.1 Software (http://markummitchell.github.io/engauge-digitizer) was used to extract time-dependent survival rates of high and low expressions groups of SNHG15. HRs with 95% CI were obtained using the methods from the previous publications [30].

### Statistical analysis

The Review Manager V 5.3 Software (Cochrane Collaboration, London, U.K.) and STATA V 12.0 Software (Stata, College Station, TX) were adopted to perform statistical analyses. The patients were classified into high and low expression groups of *SNHG15* in accordance with primary articles. OR and its 95% CI values were pooled to explore the relationship between the expression level of *SNHG15* and clinical pathological parameters including TNM stage (III+IV vs. I+II), lymph node metastasis (positive vs. negative), distant metastasis (yes vs. no), tumor size (big vs. small) and histological grade (undifferentiated/poor vs. moderate/well). For survival outcomes such as OS, DFS and PFS, HR analysis with 95% CI was performed. Heterogeneity among the studies was assessed using *I^2^* statistic and *P*-value. *I^2^* > 50% or *P*<0.05 indicated significant heterogeneity among the results of the selected studies; the random effects model was adopted in this case; and a subgroup analysis was conducted based on cancer types, HR calculation procedures and sample size to investigate the source of the heterogeneity. On the other hand, *I^2^* < 50% or *P*>0.05 indicated insignificant heterogeneity, and the fixed effects model was then adopted. A sensitivity analysis was performed to test whether the results of a single study significantly affected the overall results. The publication bias was tested by the Begg’s test.

## Results

### Characteristics of selected studies

About 187 relevant publications were initially retrieved from several databases. First, 48 duplicate records were excluded. After reading the titles and abstracts, another 97 studies were excluded. About 42 relevant studies were then identified by reading the full-texts. About 27 studies were excluded for the following reasons: (a) the association between the expression of SNHG15 and the prognosis of the patients (*n*=14) was not investigated; (b) the patients were not classified based on high and low expression groups of SNHG15 (*n*=8); or (c) no data were available for meta-analysis (*n*=5). About 15 studies were finally selected for this meta-analysis. All of these studies were conducted by researchers from China. Publication years ranged from 2015 to 2019, and the simple sizes were between 24 and 182. The expression level of *SNHG15* was quantified by the quantitative real-time PCR (qRT-PCR) method in all the selected studies. About 12 studies provided survival outcome data. About 10 cancer types were covered: glioma (*n*=1) [24], non-small cell lung cancer (NSCLC) (*n*=4) [20–23], hepatocellular carcinoma (*n*=1) [31], colorectal cancer (*n*=1) [25], breast cancer (*n*=1) [18], renal carcinoma (*n*=1) [26], gastric carcinoma (*n*=1) [19], pancreatic cancer (*n*=2) [17,32], papillary thyroid carcinoma (*n*=2) and epithelial ovarian cancer (*n*=1) [33–35]. Detailed characteristics of these studies are shown in Table 1. The scores ranged from 7 to 9, and studies with at least a total score of 6 were considered of high quality (Table 2).

**Table 1:** Basic features of the publications included in this meta-analysis (*n*=15)

Study	Year	Country	Cancer type	Patients	Reference gene	Detection method	Cut-off	HR statistics	Survival analysis	Hazard ratios(95%CI)	Follow-up (month)
Ma, X.	2019	China	NSCLC	24	GAPDH	qRT-PCR	Mean-value	Report	OS	3.30 (1.36–7.98)	60
Dong, Y.	2018	China	NSCLC	49	GAPDH	qRT-PCR	Mean-value	Survival curve	OS	1.85 (0.95–3.60)	120
									DFS	2.22 (1.17–4.21)	120
Cui, X.	2018	China	NSCLC	55	GAPDH	qRT-PCR	Mean-value	Report	OS	2.23 (1.03–4.83)	80
Jin, B.	2017	China	NSCLC	35	GAPDH	qRT-PCR	Median	Survival curve	OS	1.63 (0.56–4.74)	60
Zhang, J.	2017	China	HCC	152	GAPDH	qRT-PCR	NA	Report	OS	2.25 (1.33–3.79)	70
Liu, Y.	2018	China	PTC	136	GAPDH	qRT-PCR	Median	NA	NA	NA	60
Wu, D.	2018	China	PTC	92	GAPDH	qRT-PCR	Median	Survival curve	OS	1.89 (0.78–4.58)	60
Huang, L.	2018	China	CRC	91	GAPDH	qRT-PCR	Median	Report	OS	2.73 (1.00–7.42)	70
Qu, C.	2019	China	EOC	182	GAPDH	qRT-PCR	Mean-value	Report	OS	1.14 (1.07–1.21)	60
									PFS	1.120 (1.056–1.189)	60
Kong, Q.	2018	China	BC	58	GAPDH	qRT-PCR	Median	Survival curve	OS	1.86 (0.80–4.32)	60
Ma, Y.	2017	China	Glioma	46	GAPDH	qRT-PCR	Mean-value	Survival curve	OS	3.81 (0.34–42.69)	60
Du, Y.	2018	China	ccRcc	96	NA	qRT-PCR	Median	NA	NA	NA	NA
Chen, S.	2015	China	GC	106	GAPDH	qRT-PCR	NA	Report	OS	2.928 (1.304–6.575)	40
									DFS	2.399 (1.377–4.177)	40
Guo, X.	2018	China	PA	171	GAPDH	qRT-PCR	Mean-value	Report	OS	3.251 (1.177–6.362)	60
Ma, Z.	2017	China	PDA	48	GAPDH	qRT-PCR	NA	NA	NA	NA	NA

Abbreviations: BC, breast cancer; ccRCC, clear cell renal cell carcinoma; CRC, colorectal cancer; DFS, disease-free survival; EOC, epithelial ovarian cancer; ESCC, esophageal squamous carcinoma; GC, gastric cancer; GAPDH, glyceraldehyde-3-phosphate dehydrogenase; HCC, hepatocellular carcinoma; NA, not available; No., number; NSCLC, non-small cell lung cancer; OS, overall survival; PA, pancreatic cancer; PDA, pancreatic ductal adenocarcinoma; PFS, progress-free survival; PTC, papillary thyroid carcinoma; qRT-PCR, quantitative reverse transcription-polymerase chain reaction.

**Table 2:** Quality assessment of eligible studies Newcastle–Ottawa scale (NOS)

Author	Country	Selection				Comparability	Outcome			Total
		Adequate of case definition	Representativeness of the cases	Selection of Controls	Definition of Controls	Comparability of cases and controls	Ascertainment of exposure	Same method of ascertainment	Non-response rate	
										
										
Ma, X. (2019)	China	NA	*	*	*	**	*	*	*	8
Dong, Y. (2018)	China	*	*	*	*	**	*	*	*	9
Cui, X. (2018)	China	*	*	*	*	**	*	*	*	9
Jin, B. (2017)	China	*	*	*	*	**	*	*	*	9
Zhang, J. (2017)	China	*	*	*	*	*	*	*	*	8
Liu, Y. (2018)	China	*	*	*	*	**	*	*	NA	8
Wu, D. (2018)	China	*	*	*	*	**	*	*	*	9
Huang, L. (2018)	China	*	*	*	*	**	*	*	*	9
Qu, C. (2019)	China	*	*	*	*	**	*	*	*	9
Kong, Q. (2018)	China	*	*	*	*	**	*	*	*	9
Ma, Y. (2017)	China	*	*	*	*	**	*	*	*	9
Du, Y. (2018)	China	*	*	*	*	**	*	*	NA	8
Chen, S. (2015)	China	*	*	*	*	*	*	*	*	8
Guo, X. (2018)	China	*	*	*	*	**	*	*	*	9
Ma, Z. (2017)	China	*	*	*	*	*	*	*	NA	7

Note: NA: not available

Reasons:

1: Adequate of case definition (Ma, X. (2019)): Small number of patients in this study (*n*=24) would make Result bias to a certain extent.

2: Comparability of cases and controls (Zhang, J. (2017); Chen, S. (2015); Ma, Z. (2017)): these three studies without reporting the “cut-off value”, and reduced the comparability between the experimental group and the control group to a certain extent.

3: Non-response rate (Liu, Y. (2018); Du, Y. (2018); Ma, Z. (2017)): these three studies lack of follow-up time of patient, and we don't known whether the patient cooperates with treatment from beginning to end.

### Association between the expression level of SNHG15 and survival outcome of cancer patients

**Figure 2:**
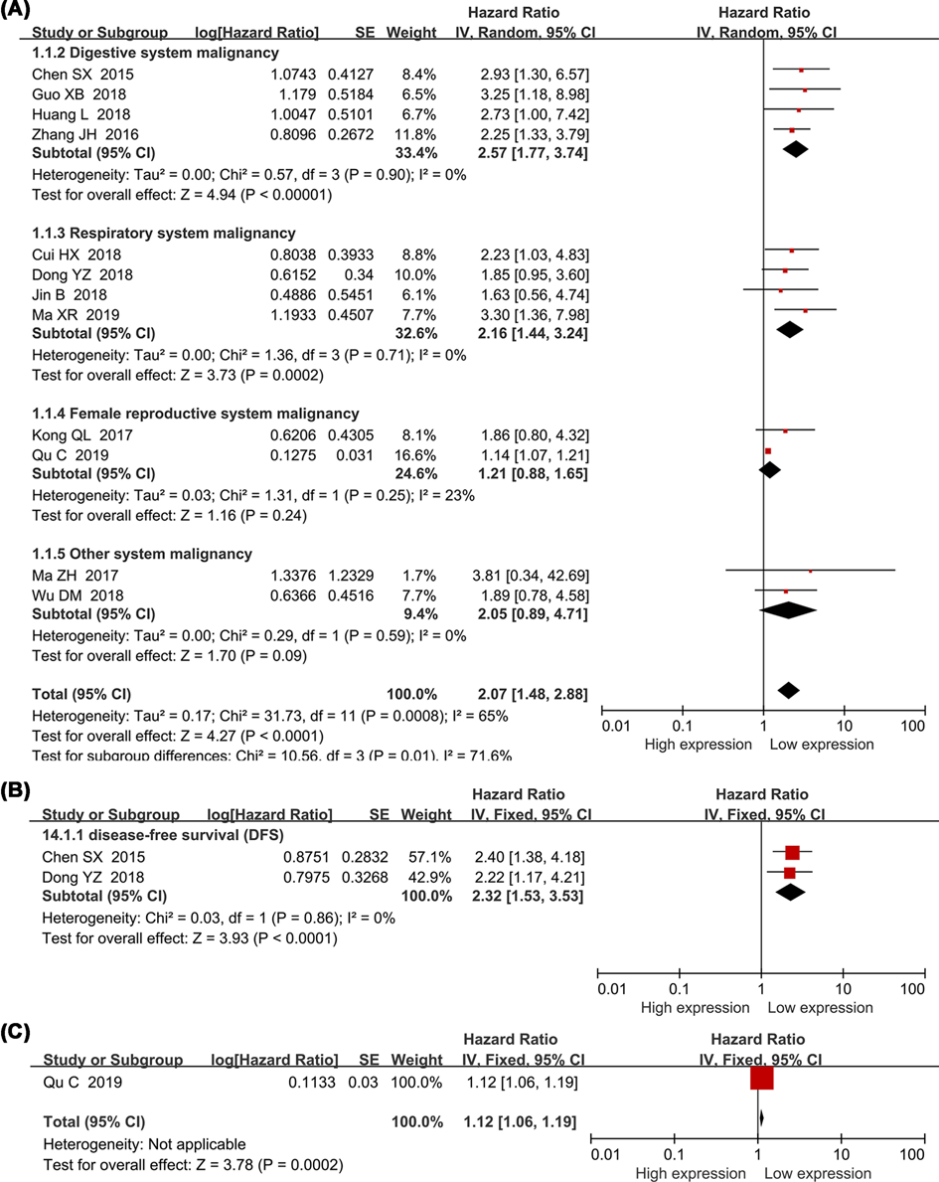
Forest plot showed the relationship between SNHG15 expression and survival prognosis in cancers OS (**A**), DFS (**B**), PFS (**C**).

Twelve studies with 1058 enrolled patients explored the association between the expression level of *SNHG15* and the overall survival in various cancers. Seven explicitly reported HR values were directly taken from respective studies (direct extraction) whereas the remaining five HR values were extracted using Engague Software (indirect extraction). Pooled HRs indicated an association between the high expression of *SNHG15* and overall survival (HR = 2.07, 95% CI, 1.48–2.88; *P*<0.0001). Take consideration of significant heterogeneity (*I^2^*= 65%, *P*=0.0008) and different expression levels of *SNHG15* in variety of tumor tissues, a subgroup analysis was nevertheless performed based on cancer types, HR extraction procedures (direct/indirect extraction), analysis methods (univariate/multivariate analysis) and sample sizes (less/more than 100 patients). A strong correlation was revealed between increasing expression of SNHG15 and poor OS in digestive system cancers (HR = 2.57, 95% CI, 1.77–3.74; *P*<0.0001), respiratory system cancers (HR = 2.16, 95% CI, 1.44–3.24; *P*=0.0002), cancers with less than 60 patients (HR = 2.04, 95% CI, 1.48–3.06; *P*<0.00001), more than 60 patients (HR = 2.04, 95% CI, 1.26–3.31; *P*=0.004), HRs extracted directly from studies (HR = 2.24, 95% CI, 1.39–3.59; *P*=0.009) or indirectly from the Kaplan–Meier curve (HR = 1.86, 95% CI, 1.24–2.81; *P*=0.003) (Figure 2A and Table 3). Analysis based on different cancer types showed that SNHG15 has the most prominent prognostic potential in glioma (HR = 3.81; 95% CI, 0.84–42.69; *P*=0.28) (Supplementary Figure S6), and the results needed to be further supported by more high quality and large sample studies. Two studies comprising 155 patients and one study comprising 182 patients were included to investigate the association between the expression of *SNHG15*, DFS and PFS (Figure 2B,C). Combining results showed that elevated expression levels of SNHG15 indicated worse DFS (HR = 2.32, 95% CI, 1.53–3.53; *P*<0.0001) and PFS (HR = 1.12, 95% CI, 1.06–1.19; *P*=0.0002; Table 3).

**Table 3:** Subgroup analysis of the pooled HRs with SNHG15 expression in patients with cancer

Subgroup analysis	No. of studies	No. of patients	Pooled HR(95%CI)	*P*	Heterogeneity		Model
					*I*^2^(%)	*P*-value	
**OS**	12	1058	2.07 (1.48–2.88)	<0.0001	65	0.0008	Random
**Type of analysis**							
Univariate analysis	8	462	2.20 (1.61–2.99)	<0.00001	0	0.94	Fixed
Multivariate analysis	4	596	1.93 (1.08–3.47)	<0.00001	77	0.004	Random
**Tumor type**							
Digestive system cancer	4	520	2.57 (1.77–3.74)	<0.00001	0	0.9	Fixed
Respiratory system malignancy	4	163	2.16 (1.44–3.24)	<0.0002	0	0.71	Fixed
Female reproductive system malignancy	2	240	1.21 (0.88–1.65)	0.24	23	0.25	Fixed
Other system malignancy	2	115	2.05 (0.89–4.71)	0.09	0	0.59	Fixed
**HR estimation method**							
Indirectly	5	257	1.86 (1.24–2.81)	0.003	0	0.98	Fixed
Directly	7	781	2.24 (1.39–3.59)	0.009	77	0.002	Random
**number of patients**							
More than 60	6	791	2.04 (1.26–3.31)	0.004	74	0.002	Random
Less than 60	6	267	2.13 (1.48–3.06)	<0.00001	0	0.89	Fixed
**Cut-off**							
Median-value	5	325	1.94 (1.32–2.84)	0.0007	0	0.96	Fixed
Mean-value	5	455	2.12 (1.13–3.97)	0.02	70	0.009	Random
NA	2	258	2.43 (1.57–3.77)	<0.0001	0	0.59	Fixed
**Quality scores**							
Score = 9	9	779	1.79 (1.27–2.52)	0.0008	50	0.05	Random
Score < 9	3	279	2.58 (1.74–3.83)	<0.0001	0	0.72	Fixed
**DFS**	2	155	2.32 (1.53–3.53)	<0.0001	0	0.86	Fixed
**PFS**	1	182	1.12 (1.06–1.19)	0.0002	-	-	-

Abbreviations: DFS, disease-free survival; OS, overall survival; PFS, progression-free survival. Random, random-effect model; Fixed, fixed-effects model; directly, HR was extracted directly from the primary articles; indirectly, HR was extracted indirectly from the primary articles.

### Association between the expression level of SNHG15 and TNM stage

**Figure 3:**
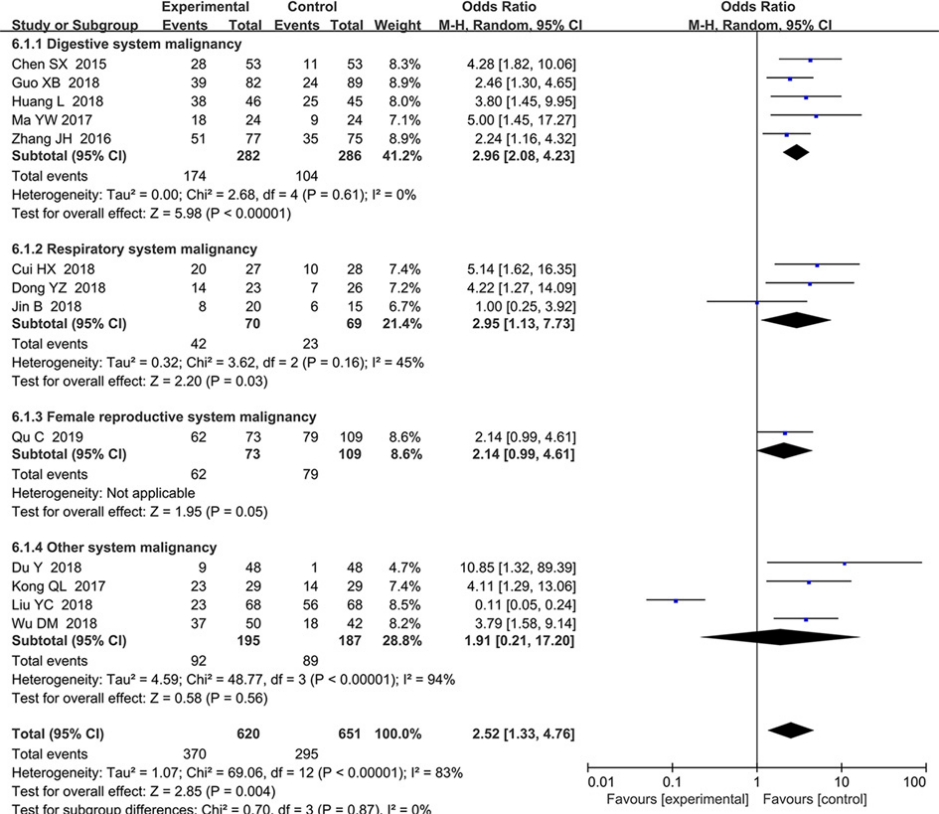
Forest plot about the relationship between the expression of SNHG15 and TNM stage

About 13 studies comprising 1271 patients were included to explore the association between the expression level of SNHG15 and TNM stage (Figure 3). The elevated expression of *SNHG15* was associated with advanced TNM stage (HR = 2.52, 95% CI, 1.33–4.76; *P*=0.004). A subgroup analysis further suggested that high expression of SNHG15 meant advanced TNM stage in digestive system cancers (HR = 2.96, 95% CI, 2.08–4.43; *P*=0.004), respiratory system cancers (HR = 2.95, 95% CI, 1.13–7.73; *P*=0.03) and female reproductive system cancers (HR = 2.61, 95% CI, 1.38–4.95; *P*=0.003), yet a irrelevant association between the expression level of SNHG15 and TNM stage in other systems was also observed (HR = 1.50, 95% CI, 0.09–26.42; *P*=0.78) (detail in Table 4). A random effects model was applied due to high heterogeneity level (*I^2^*= 83, *P*<0.0001).

**Table 4:** Pool effects of Clinicopathologic characteristics in cancer patients with abnormal SNHG15 expression

Clinicopathological characteristics	No. of studies	No. of patients	Odds ratio (95% CI)		Heterogeneity	
			Fixed	Random	*I*^2^(%)	*P*-value
Age (old vs. young)	13	1271	0.98 (0.78–1.22)	0.98 (0.78–1.23)	0.0	0.680
gender (female vs. male)	11	1031	1.07 (0.83–1.37)	1.05 (0.78–1.41)	23	0.220
TNM (III+IV vs. I+II)	13	1271	1.98 (1.57–2.51)	2.52 (1.33–4.76)	83.9	0.000
Digestive system	5	568	2.97 (2.09–4.23)	2.96 (2.08–4.23)	0	0.61
Respiratory system	3	139	3.04 (1.52–6.09)	2.95 (1.13–7.73)	45	0.16
Female reproductive system	1	182	2.14 (0.99–4.61)	2.14 (0.99–4.61)	-	-
Other system malignancy	4	382	0.96 (0.64–1.45)	1.91 (0.21–17.20)	94	0.000
LNM (present vs. absent)	11	937	2.09 (1.65–2.64)	2.41 (0.99–5.87)	87	0.000
Digestive system	4	416	3.00 (2.00–4.51)	2.98 (1.98–4.49)	0	0.450
Respiratory system	3	139	3.40 (1.67–6.91)	3.39 (1.66–6.93)	0	0.67
Other system malignancy	4	382	0.71 (0.46-1.09)	1.39 (0.09–20.34)	95	0.000
Tumor size (big vs. small)	8	761	1.48 (1.11–1.97)	2.06 (1.03–4.13)	79.0	0.000
Digestive system	3	371	1.24 (0.82–1.88)	1.47 (0.56–3.88)	77.0	0.010
Respiratory system	2	104	4.63 (2.02–10.66)	4.63 (2.01–10.66)	0	0.81
Other system malignancy	3	286	1.26 (0.79–2.02)	1.88 (0.41–8.59)	87.0	0.000
Histological grade	7	701	2.62 (1.90–3.59)	2.59 (1.84–3.64)	9.0	0.360
Digestive system	5	570	2.63 (1.84–3.76)	2.64 (1.84–3.77)	0.0	0.550
Non-digestive system	2	131	2.56 (1.27–5.15)	2.05 (0.47–-9.04)	72.0	0.060
DM (present vs. absent)	5	521	1.66 (1.02–2.72)	1.64 (0.40–6.74)	73.0	0.005
Digestive system	2	197	5.05 (2.15–11.85)	5.10 (2.17–11.96)	0.0	0.430
Non-digestive system	3	324	0.83 (0.43–1.61)	0.66 (0.03–12.65)	80.0	0.007
Invasion depth (T3+T4/T1+T2)	3	349	4.13 (2.55–6.67)	3.60 (0.95–13.66)	84	0.002
smoking (smoker vs. non-smoker)	2	84	1.03 (0.24–2.54)	1.15 (0.26–5.11)	59.0	0.120

Abbreviations: DM, distant metastasis; LNM, lymph node metastasis. Random, random-effect model; TNM, TNM stage; Fixed, fixed-effect model.

### Association between the expression level of SNHG15 and lymph node metastasis (LNM)

Eleven studies comprising 937 patients were included to assess the association between the expression level of SNHG15 and lymph node metastasis. The combined results indicated a significantly positive correlation between the high expression of SNHG15 and positive lymph node metastasis (HR = 2.41, 95%CI, 0.99–5.87; *P*=0.05). The random effects model was applied and a subgroup analysis was conducted due to significant heterogeneity (*I^2^*= 87, *P*<0.0001), an obvious association between the high expression of SNHG15 and distant lymph node metastasis was revealed in digestive system cancers (HR = 2.98, 95% CI, 1.98–4.49; *P*<0.00001), respiratory system cancers (HR = 3.39, 95% CI, 1.66–6.93; *P*=0.0008), female reproductive system cancers (HR = 4.41, 95% CI, 1.22–16.0; *P*=0.02). But an indistinct relationship was also observed in other system malignancy (HR = 0.94, 95% CI, 0.03–31.80; *P*=0.97; Table 4).

### Association between SNHG15 expression level and other clinicopathologic parameters

A strong positive correlation between the high expression of SNHG15 and poor histologic grade (OR = 2.62, 95% CI, 1.90–3.59; *P*<0.00001), bigger tumor sizes (OR = 2.06, 95% CI, 1.03–4.13; *P*=0.04) was uncovered, and the irrelevance between the expression of SNHG15 and the depth of invasion (HR = 3.60, 95% CI, 0.95–13.66; *P*=0.06), distant metastasis (OR = 1.64, 95% CI, 0.40–6.74; *P*=0.49), smoking history (OR = 1.15, 95% CI, 0.26–5.11; *P*=0.85), age (OR = 0.98, 95% CI, 0.78–1.28; *P*=0.84) and gender (OR = 1.07, 95% CI, 0.83–1.37; *P*=0.61) were also observed (detail in Table 4).

### Sensitivity analysis and publication bias

**Figure 4:**
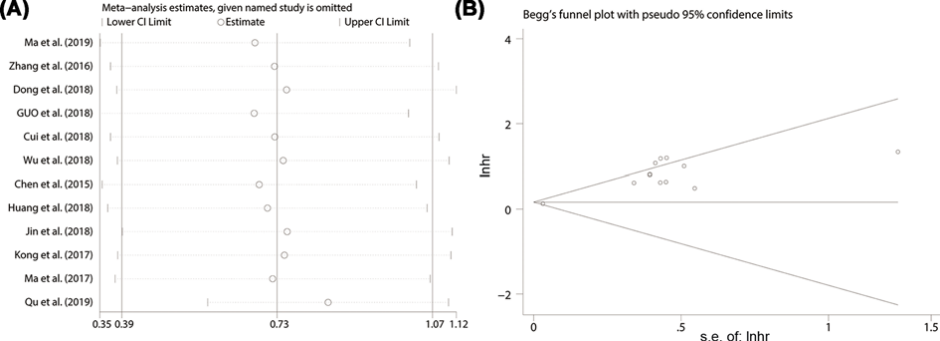
Sensitivity analysis and Funnel plot for the expression of SNHG15 with OS in various cancers HR, hazard ratio; CI, confidence interval

The sensitivity analysis was performed to test whether the result of a single study exerted a significant effect on the overall results. None of the included studies had a significant influence on the overall results (Figure 4A). The potential publication bias was estimated by Begg’s test, as exhibited in Figure 4B. Apart from the histological grade (*P*r > |*z*| = 0.016) and tumor size (*P*r > |*z*| = 0.016), unobvious publication bias was found for the included studies of OS (*P*r > |*z*| = 0.945), RFS (*P*r > |*z*| = 0.317), LNM (*P*r > |*z*| = 0.755), TNM stage (*P*r > |*z*| = 0.360), DM (*P*r > |*z*| = 1.00), depth of invasion (*P*r > |*z*| = 0.602), smoking (*P*r > |*z*| = 0.317), age (*P*r > |*z*| = 0.760) and gender (*P*r > |*z*| = 0.276). Consequently, there was no significant publication bias in this meta-analysis.

### Conformation of the results in TCGA cohort

**Figure 5:**
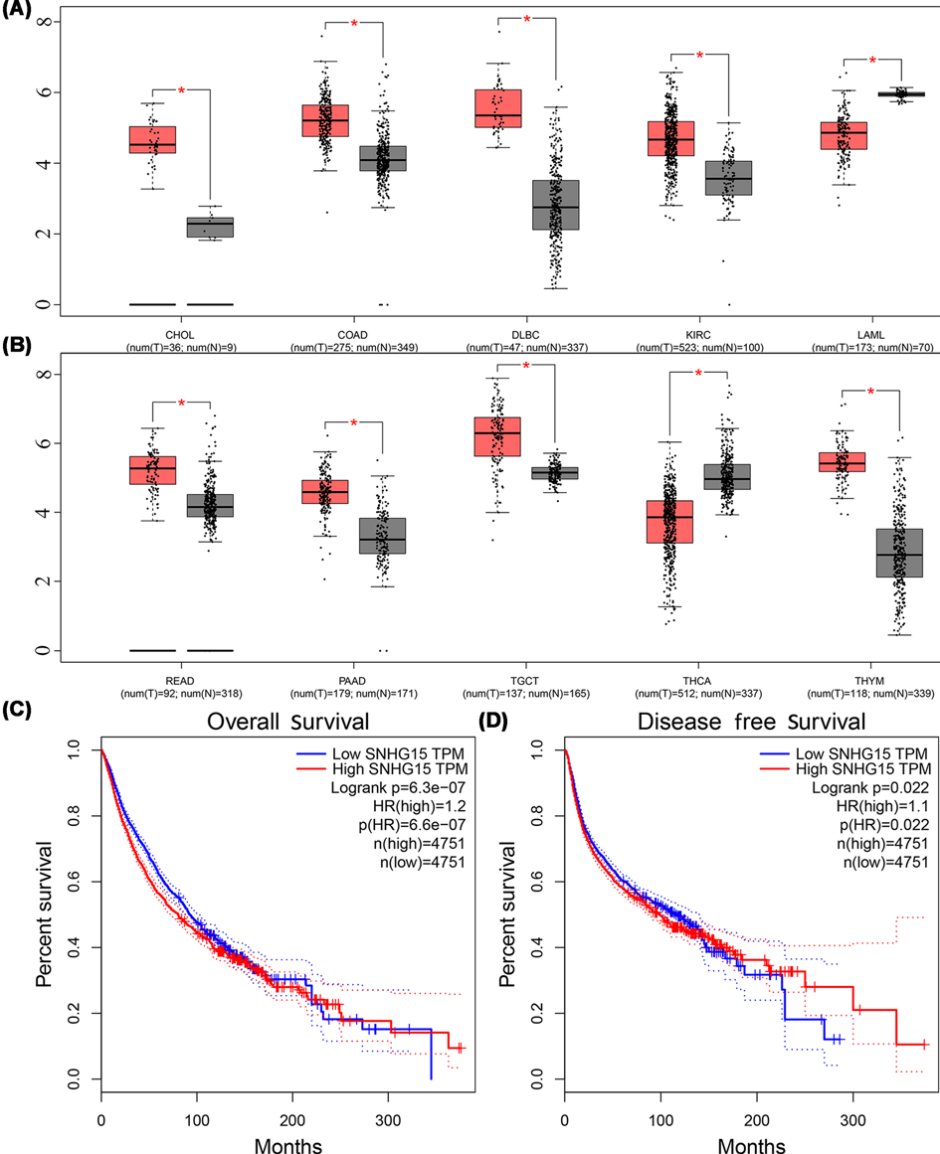
The expression levels and survival prognosis of SNHG15 in cancers via accessing the GEPIA cohort cholangiocarcinoma, colonadenocarcinoma, lymphoid neoplasm diffuse large B-cell lymphoma, kidney renal clear cell carcinoma, acute myeloid leukemia (**A**). pancreatic adenocarcinoma, rectum adenocarcinoma, testicular germ cell tumors, thyroid carcinoma and thymoma were revealed in (**B**). Survival plots in the GEPIA cohort via merging the expression data of SNHG15 and OS (**C**) (*n*=9502). Survival plots in the GEPIA cohort via merging the expression data of SNHG15 and DFS (**D**) (*n*=9502).

**Figure 6:**
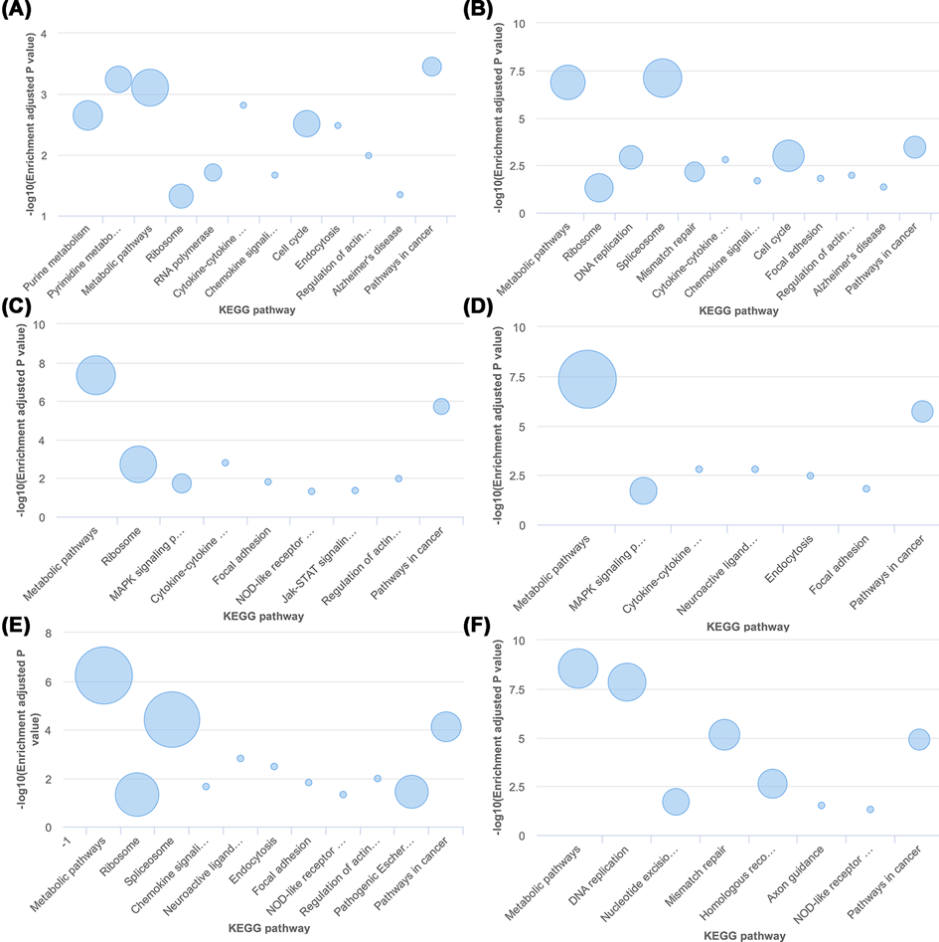
SNHG15 involved in a serious of cellular biological role via accessing the TCGA cohort gastric cancer (**A**), breast cancer (**B**), colorectal cancer (**C**), NSCLC cancer (**D**), liver cancer (**E**) and esophageal squamous cell carcinoma (**F**)

To further verify our conclusions, TCGA cohort was explored to investigate the expression of SNHG15 in multiple types of tumor tissues, the abnormal expression of SNHG15 was revealed in cholangiocarcinoma, colon adenocarcinoma, lymphoid neoplasm diffuse large B-cell lymphoma, kidney renal clear cell carcinoma, acute myeloid leukemia (Figure 5A), pancreatic adenocarcinoma, rectum adenocarcinoma, testicular germ cell tumors and thyroid carcinoma and thymoma (Figure 5B). Survival plots in the GEPIA cohort (http://gepia.cancer-pku.cn/) via merging the expression data of SNHG15 and OS and DFS data of malignancies from all of the TCGA databases were also adopted, containing 9502 patients who were divided into high or low expression groups based on the expression of SNHG15, and the results showed that the elevated expression of SNHG15 predicted unsatisfactory OS (Figure 5C) and DFS (Figure 5D), which supported our results in this meta-analysis. In addition, by accessing KEGG database, SNHG15 was uncovered involving in the occurrence and development of cancers including gastric cancer, breast cancer, NSCLC, colorectal cancer, liver cancer and esophageal squamous cell carcinoma and so on (Figure 6A–F), involving in the cell cycle in breast cancer and gastric cancer (Figure 6A,B).

## Discussion

With improvements in medicine, diverse treatment methods including chemotherapy, radiotherapy, immunotherapy and targeted therapy were adopted in cancer treatment [36], the survival outcomes and the quality of life were improved in the last decade. Yet these achievements are insignificant as the conditions of a vast majority of malignant patients who are still irreversible with continuous deterioration. The 5-year overall survival and progression-free survival rates are still unsatisfactory as well. Cancer occurrence and progression are not simply due to a single gene mutation or chromosomal aberration and various molecules are involved in the process as a result of the influence of individual and environmental factors [37,38]. In recent years, micro-RNA, lncRNA and circular RNA attracted the interests of the cancer researchers greatly [39]. For a long time, non-coding RNA had been considered to perform no apparent function even though it represented a large portion of transcription products [40]. Evidences from recent years indicated that non-coding RNA was involved in multiple cellular processes, including transportation, epigenetic regulation within the nucleus, variable shear and cell cycle regulation [41]. The abnormal expression of non-coding RNA was reported to be closely related to diseases, such as cancer, cardiovascular disease, inflammatory bowel disease and metabolic disease [42].

lncRNA *SNHG15* was first discovered in melanoma. Previous studies reported high expression levels of *SNHG15* in various cancer types, such as gastric cancer, colorectal cancer, osteosarcoma, glioma, lung cancer and breast cancer, and similar conclusions were confirmed by the evidences from TCGA cohort. *SNHG15* also influenced a series of biological behaviors of tumor cells, and could therefore serve as a potential indicator for cancer prognosis. Thus, a meta-analysis was performed here to assess the prognostic values of SNHG15 in cancers.

**Figure 7:**
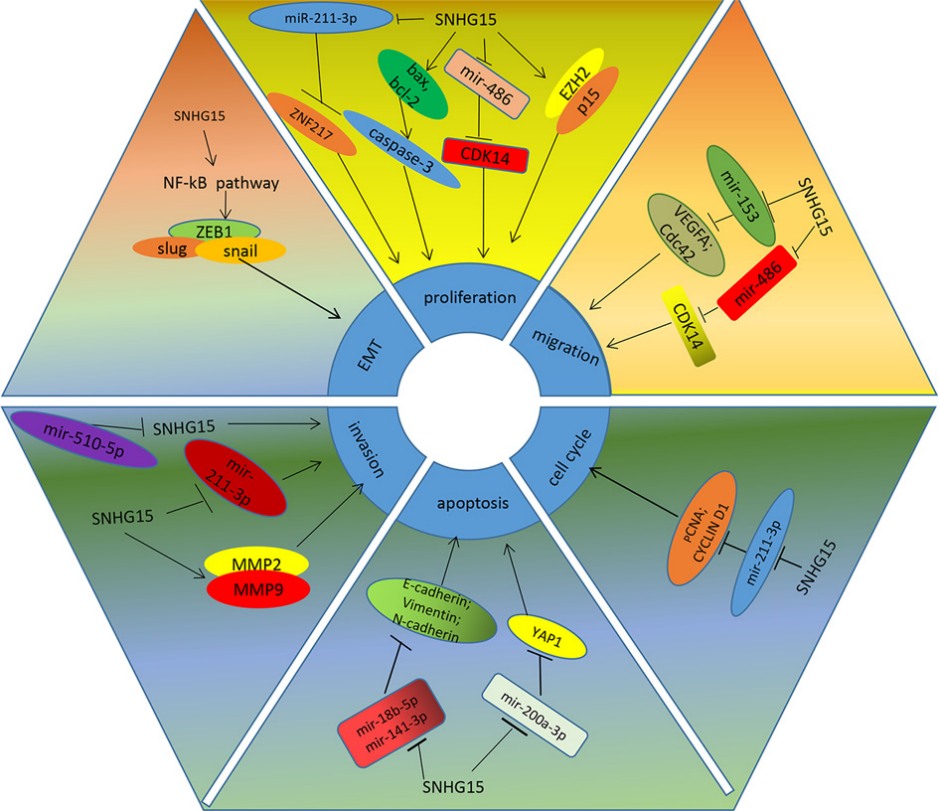
The summary of underlying molecular mechanisms of abnormal SNHG15 expression in the development of cancer

Several researchers tried to explore the molecular mechanism of lnc*SNHG15* in the development of various cancer types (Table 5 and Figure 7) [20,26]. Chen et al. demonstrated that *SNHG15* could regulate cell proliferation by acting on MMP (matrix metallopeptidase)2 and (matrix metallopeptidase 9) MMP9 in gastric cancer cells, but the potential mechanisms of actions of SNHG15 and MMP2/MMP9 have not been explored [19]. Ma et al. reported that *SNHG15* regulated the expression of zinc finger protein 217 (ZNF217) through sponging miR-211-3p, and thereby contributed to proliferation and migration of NSCLC [23]. Jin et al. found out that SNHG15 could promote the growth, invasion and migration of NSCLC cells by regulating cyclin-dependent kinase 14 (CDK14) expression via sponging miR-486 [22]. Dong et al. verified that *SNHG15* inhibited apoptosis and drives invasion and migration of NSCLC cells via interacting with MMP-2, MMP-9, Bax, Bcl-2 and poly ADP-ribose polymerase (PARP) [21]. Ma et al. reported that SNHG15 induced the cell proliferation and tumorigenesis of pancreatic cancer cells by negatively regulating p15 and krueppel-like factor 2 (KLF2) expression, and the up-regulated expression of p15 and KLF2 could partly reversed the cancer-promoting effect of SNHG15 [17]. Du et al. suggested that in renal cell carcinoma cells, SNHG15 could activate the NF-κB signaling pathway and induce epithelial–mesenchymal transformation (EMT) through up-regulating the level of Vimentin and N-cadherin expression and down-regulating the level of E-cadherin, promoting the proliferation, migration and invasion of cancer cells [26]. Kong et al. confirmed that SNHG15 regulated the proliferating cell nuclear antigen (PCNA), cyclin D1; the expression *Caspase-3* by sponging miR-211-3p, and thereby induce EMT of breast cancer cells [18]. Wu et al. discovered that *SNHG15* regulated YAP1-Hippo signaling pathway by sponging miR-200a-3p, inducing the growth and migration of papillary thyroid carcinoma cells [34]. On the contrary, Liu et al. indicated that the increasing expression of SNHG15 inhibited the proliferation, migration and tumorigenesis of thyroid tumor cells; unfortunately, they did not explore the detail molecular mechanisms [33]. In the colorectal cancer, Saeinasab et al. demonstrated that MYC protein increased the expression of SNHG15 through binding to the transcription start point of SNHG15, and the high expression of SNHG15 activated the apoptosis induced factor (AIF) protein, thus promoting the proliferation, invasion and chemotherapy resistance in colorectal cancer [43]. Jiang et al. uncovered that the up-regulated expression of SNHG15 could increase the resistance of colon cancer cells to 5-FU by acting on Slug and inhibited its degradation [44]. Ma et al. reported that *SNHG15* regulated the vascular endothelial growth factor A (VEGFA) and the expression of Cdc42 by sponging miR-153, contributing to the occurrence and development of glioma [24]. In the hepatocellular carcinoma cells, SNHG15 served as the competitive endogenous RNA, increased the expression of E-box Binding Homeobox 2 (EZB2) and E2F Transcription Factor 3 (E2F3) via sponging miR-141-3p and mediating the cell proliferation, G0/G1 arrest and cell invasion [45]. SNHG15 was also reported promoting the migration, invasion, proliferation and chemotherapy resistance of epithelial ovarian cancer cells, but detail molecular biology mechanism is still unclear [35]. In colorectal cancer (CRC) cells, the elevated expression of SNHG15 was observed to be contributed to the cell proliferation, invasion and 5-Fu resistance, but the oncological effects of SNHG15 in CRC could be partly weaken with the high expression of miR-141 [46]. In the Prostate cancer cells, SNHG15 was uncovered to participate in the EMT progress as down-regulated E-cadherin and up-regulated of N-cadherin expression [44]. We also uncovered that SNHG15 was involved in the cell cycle in breast cancer and gastric cancer via exploring the KEGG database, and the conclusion was confirmed by Kong et al. who reported that the low expression of SNHG15 decreased Phase S of breast cancers by down-regulating the bax and cycle D1 expression and up-regulating cleaved expression of caspase 3 [18], and now there is still no literature to explore the correlation between abnormal expression of SNHG15 and cell cycle in gastric cancer cells.

**Table 5:** Regulation mechanism of SNHG15 involved in various cancers

Cancer type	Expression	Micro-RNAs	Targets	Functions	Reference
Non-small cell lung cancer	Up-regulation	miR-211-3p	ZNF217	Promoted the proliferation and migration	[23]
	Up-regulation	–	MMP-2; MMP-9; Bax; Bcl-2; PARP	Invasion and metastasis; apoptosis	[21]
	Up-regulation	miR-211-3p	–	Proliferation and migration	[20]
	Up-regulation	miR-486	CDK14	Cell proliferation and apoptosis; cell cycle arrest	[22]
Papillary thyroid carcinoma	Up-regulation	–	–	Proliferation, migration and invasion	[33]
	Up-regulation	miR-200a-3p	YAP1-Hippo signaling pathway	Cell growth and migration; apoptosis	[34]
Colorectal cancer	Up-regulation	NA	NA	Cell migration and invasion	[25]
	Up-regulation	NA	MYC protein, AIF protein	Proliferation, invasion and chemotherapy resistance	[43]
	Up-regulation	miR-141	miR-141/SIRT1/Wnt/β-catenin axis	5-Fu resistance	[46]
	Up-regulation	NA	Slug	Increase the resistance to 5-FU	[44]
HCC	Up-regulation	miR-141-3p	EZB2, E2F3	Cell proliferation, G0/G1 arrest and cell invasion	[45]
Epithelial ovarian cancer	Up-regulation	NA	NA	Migration, invasion, proliferation and chemoresistance	[35]
Breast cancer	Up-regulation	miR-211-3p	PCNA, CYCLIN D1; Caspase-3; Bax	Proliferation, apoptosis, epithelial–mesenchymal transition	[18]
Malignant glioma	Up-regulation	miR-153	VEGFA, Cdc42	Cell proliferation, migration and tube formation	[24]
Renal cell carcinoma	Up-regulation	–	NF-κB signaling pathway	Proliferation and EMT	[26]
Gastric cancer	Up-regulation	–	MMP2/MMP9	Cell migration and invasion	[19]
Pancreatic ductal adenocarcinoma	Up-regulation	–	–	Tumor differentiation, lymph node metastasis and tumor stage	[25]
	Up-regulation	–	p15; KLF2; EZH2	Cell proliferation, cycle and migration	[17]

Abbreviations: 5-FU, 5-fluorouracil; AIF, apoptosis-induced factor; Bcl-2, B-cell lymphoma 2; EMT, epithelial–mesenchymal transition; EZH2, enhancer of zeste homolog 2; KLF2, Kruppel-like factor 2; MMP-2, matrix metalloproteinase 2; MMP-9, matrix metalloproteinase 9; PARP, poly ADP-ribose polymerase; PCNA, proliferating cell nuclear antigen; VEGFA, vascular endothelial growth factor A; ZEB1, zinc fnger E-box-binding homeobox 1; ZNF217, Zinc finger protein 217.

This research manifested that the high expression levels of *SNHG15* were correlated with poor clinicopathological characteristics such as advanced TNM stage, earlier lymph node (Supplementary Figure S1) and distant metastasis (Supplementary Figure S2A), worse pathological grade (Supplementary Figure S2B), larger tumor size (Supplementary Figure S3A) and deeper local invasion (Supplementary Figure S3B), and no association with smoking (Supplementary Figure S3C), age (Supplementary Figure S4A) and gender (Supplementary Figure S4B) was found. The Begg’s test was performed and insignificant publication bias were observed (Supplementary Figure S5), further confirmed the stability of the results and the homogeneity between enrolled studies. In addition, high expression level of SNHG15 was associated with shorter overall survival, progression-free survival and recurrence-free survival. Given the obvious publication bias (Figure 4B) and significant heterogeneity (Figure 2), subgroup analysis was conducted based on different cancer types, data extraction methods and number of cases in included studies. The results showed that high expression level of SNHG15 was associated with shorter overall survival in digestive, respiratory and female reproductive system cancers, and supported the positive association between high expression level of SNHG15 and poor prognosis in various cancer types. In order to further confirm the worse prognostic role of SNHG15 in cancers, we also accessed TCGA cohort, and the high expression of SNHG15 in the most of tumor tissues and bad prognostic factor of SNHG15 in various cancers was identified.

The present study is the first meta-analysis exploring the connection between the abnormal expression of *SNHG15* and cancer prognosis. Adequate evidences were collected and a comprehensive subgroup analysis was conducted to fully investigate the prognostic value of *SNHG15* in various cancer types. A significant correlation was revealed between the high expression level of SNHG15 and poor prognosis in various cancer types. Detailed molecular biological mechanisms between the abnormal expression of SNHG15 and the development of cancers were also discussed and summarized. However, there were several limitations as well. First, data from only ten cancer types were included, and therefore the conclusions may not be representative of all types of cancers. Second, the number of studies per cancer type or the sample sizes for several studies was still less, it would be better to perform larger samples, high quality and multi-center studies to further support the conclusions of this meta-analysis. Third, statistical results might be biased since HR values from several studies were indirectly extracted by using Engauge Digitizer Software. Fourth, the patients included in 14 out of 15 selected studies were from China, and therefore the results might represent only the Chinese population.

## Conclusion

SNHG15 could serve as the competitive endogenous RNA, interacting with miR-211-3p, miR-200a-3p, miR-153, miR-141-3p, miR-141 and so on, directly or indirectly acting on downstream signaling pathway, promoting the cell proliferation, migration, invasion or cycle arrest of most cancers, the high expression of SNHG15 manifested worse cancer prognosis, especially in the glioma. And SNHG15 might be served as underlying therapeutic target and promising prognostic biomarker.

## Highlights

1. Small Nucleolar RNA Host Gene 15 (*SNHG15*) was observed with a high expression in the most of tumor tissues.
2. The high expression of the SNHG15 manifested bad cancer prognosis.
3. SNHG15 could be served as a potential therapeutic target and prognostic marker

## Supplementary Material

Supplementary Figures S1-S6

## Data Availability

All data generated or analyzed during this study are included in this published article or are available from the corresponding author on reasonable request.

## Abbreviations

BC: breast cancer
Bcl-2: B-cell lymphoma 2
ccRCC: clear cell renal cell carcinoma
CRC: colorectal cancer
DFS: disease-free survival
directly: HR was extracted directly from the primary articles
DM: distant metastasis
EMT: epithelial–mesenchymal transition
EOC: Epithelial Ovarian Cancer
ESCC: Esophageal Squamous Cell Carcinoma
Fixed: Fixed-effect model
GC: gastric cancer
HCC: hepatocellular carcinoma
indirectly: HR was extracted indirectly from the primary articles
LNM: lymph node metastasis
MMP-2: matrix metalloproteinase 2
MMP-9: matrix metalloproteinase 9
NA: not available
NSCLC: non-small cell lung cancer
OS: overall survival
PA: pancreatic cancer
PARP: poly ADP-ribose polymerase
PCNA: proliferating cell nuclear antigen
PDA: pancreatic ductal adenocarcinoma
PFS: progression-free survival
PTC: papillary thyroid Carcinoma
qRT-PCR: quantitative reverse transcription-polymerase chain reaction
Random: random-effect model
TNM: TNM stage
VEGFA: vascular endothelial growth factor A
ZEB1: zinc fnger E-box-binding homeobox 1
ZNF217: Zinc finger protein 217
