# Supplementary material for: High lncSNHG15 expression may predict poor cancer prognosis: a meta-analysis based on the PRISMA and the bio-informatics analysis
Source: Biosci Rep. 2020 Jul 17;40(7):BSR20194468. doi: 10.1042/BSR20194468 (PMC7369394; doi:10.1042/BSR20194468)
Supplement: Supplementary Figures S1-S6 [file BSR-2019-4468_supp.pdf]

Study or Subgroup	Experimental Events	Experimental Total	Control Events	Control Total	Weight	Odds Ratio M-H, Random, 95% CI
**7.1.1 Digestive system malignancy**						
Chen SX 2015	29	53	17	53	10.2%	2.56 [1.16, 5.64]
Guo XB 2018	42	82	23	89	10.4%	3.01 [1.58, 5.73]
Huang L 2018	30	46	20	45	10.0%	2.34 [1.01, 5.46]
Ma YW 2017	20	24	9	24	8.8%	8.33 [2.15, 32.30]
**Subtotal (95% CI)**		**205**		**211**	**39.4%**	**2.98 [1.98, 4.49]**
Total events	121		69			
Heterogeneity: Tau² = 0.00; Chi² = 2.67, df = 3 (P = 0.45); I² = 0%						
Test for overall effect: Z = 5.23 (P < 0.00001)						
**7.1.2 Respiratory system malignancy**						
Cui HX 2018	21	27	12	28	9.3%	4.67 [1.44, 15.13]
Dong YZ 2018	15	23	9	26	9.3%	3.54 [1.09, 11.51]
Jin B 2018	14	20	8	15	8.7%	2.04 [0.51, 8.23]
**Subtotal (95% CI)**		**70**		**69**	**27.2%**	**3.39 [1.66, 6.93]**
Total events	50		29			
Heterogeneity: Tau² = 0.00; Chi² = 0.80, df = 2 (P = 0.67); I² = 0%						
Test for overall effect: Z = 3.35 (P = 0.0008)						
**7.1.3 Female reproductive system malignancy**						
Kong QL 2017	25	29	17	29	9.0%	4.41 [1.22, 16.00]
**Subtotal (95% CI)**		**29**		**29**	**9.0%**	**4.41 [1.22, 16.00]**
Total events	25		17			
Heterogeneity: Not applicable						
Test for overall effect: Z = 2.26 (P = 0.02)						
**7.1.4 Other system malignancy**						
Du Y 2018	1	48	0	48	4.5%	3.06 [0.12, 77.09]
Liu YC 2018	11	68	51	68	10.0%	0.06 [0.03, 0.15]
Wu DM 2018	41	50	19	42	9.8%	5.51 [2.15, 14.16]
**Subtotal (95% CI)**		**166**		**158**	**24.4%**	**0.94 [0.03, 31.80]**
Total events	53		70			
Heterogeneity: Tau² = 8.77; Chi² = 48.62, df = 2 (P < 0.00001); I² = 96%						
Test for overall effect: Z = 0.03 (P = 0.97)						
**Total (95% CI)**		**470**		**467**	**100.0%**	**2.41 [0.99, 5.87]**
Total events	249		185			
Heterogeneity: Tau² = 1.87; Chi² = 79.82, df = 10 (P < 0.00001); I² = 87%						
Test for overall effect: Z = 1.94 (P = 0.05)						
Test for subgroup differences: Chi² = 0.82, df = 3 (P = 0.84), I² = 0%						

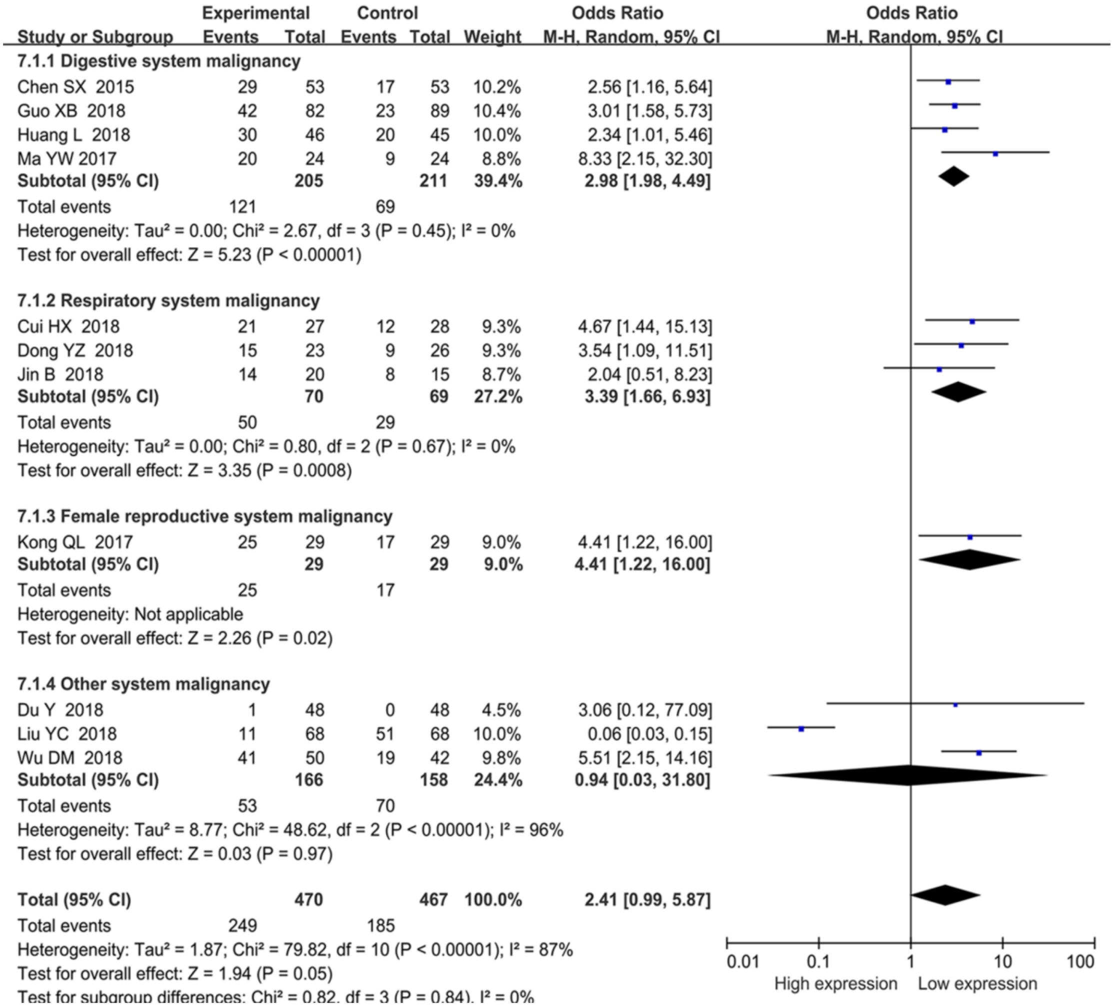

Fig. S1. Forest plot about the relationship between SNHG15 expression and the lymph node metastasis.

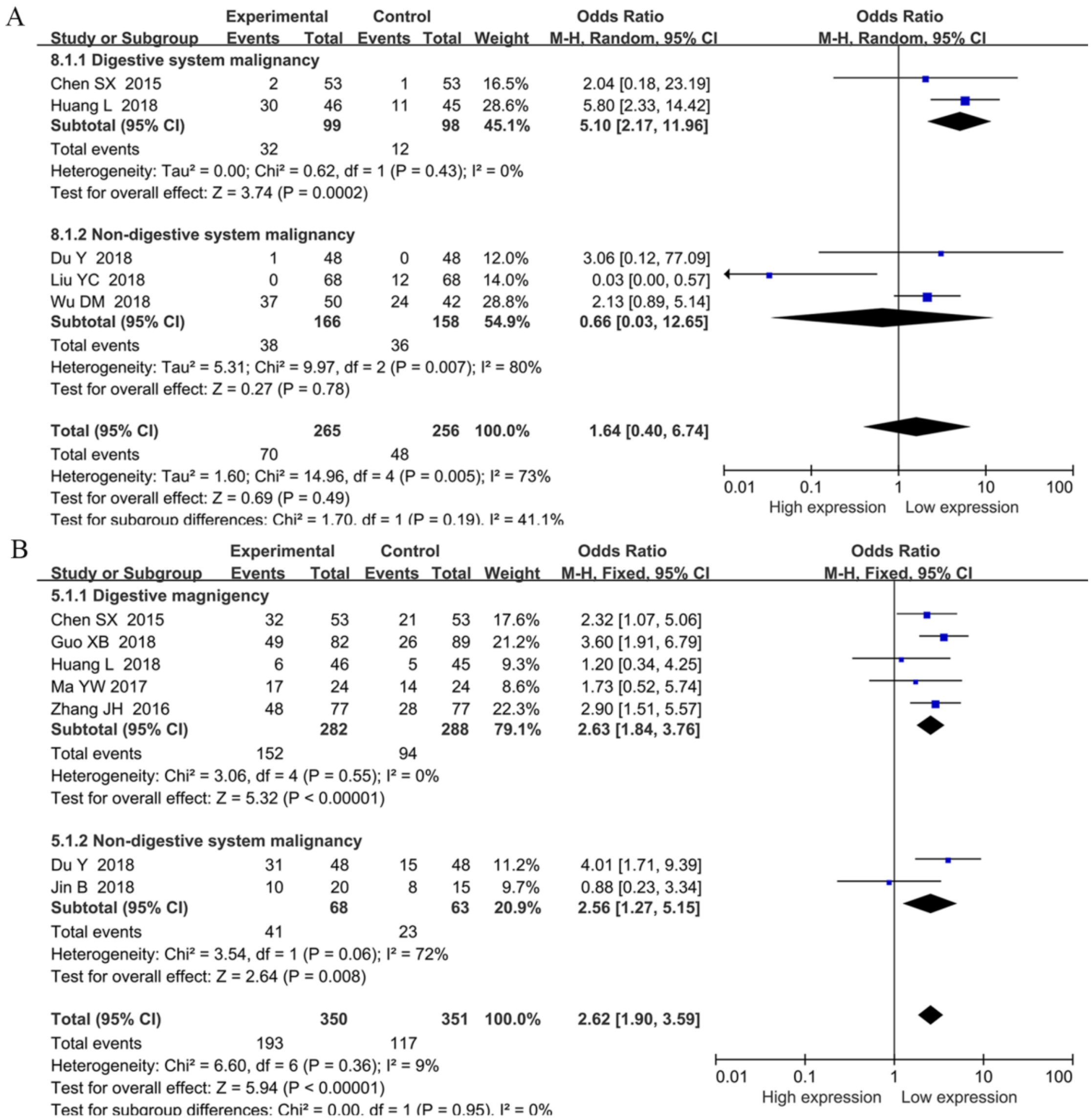

Fig. S2. Forest plot about the relationship between SNHG15 expression and distant metastasis (A) and histologic grade (B).

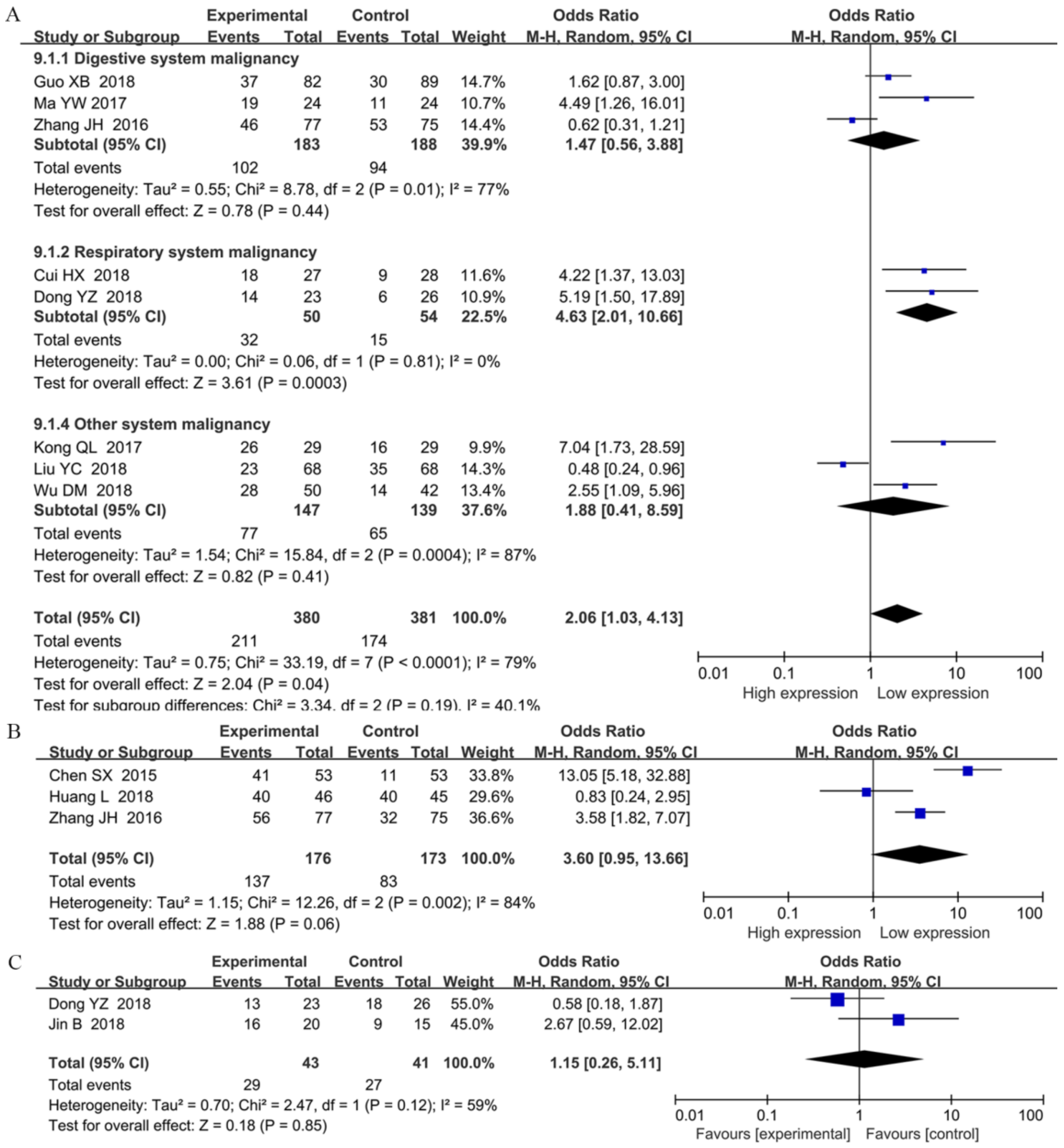

Fig. S3. Forest plot about the relationship between SNHG15 expression and depth of invasion (A), tumor size (B) and smoking (C).

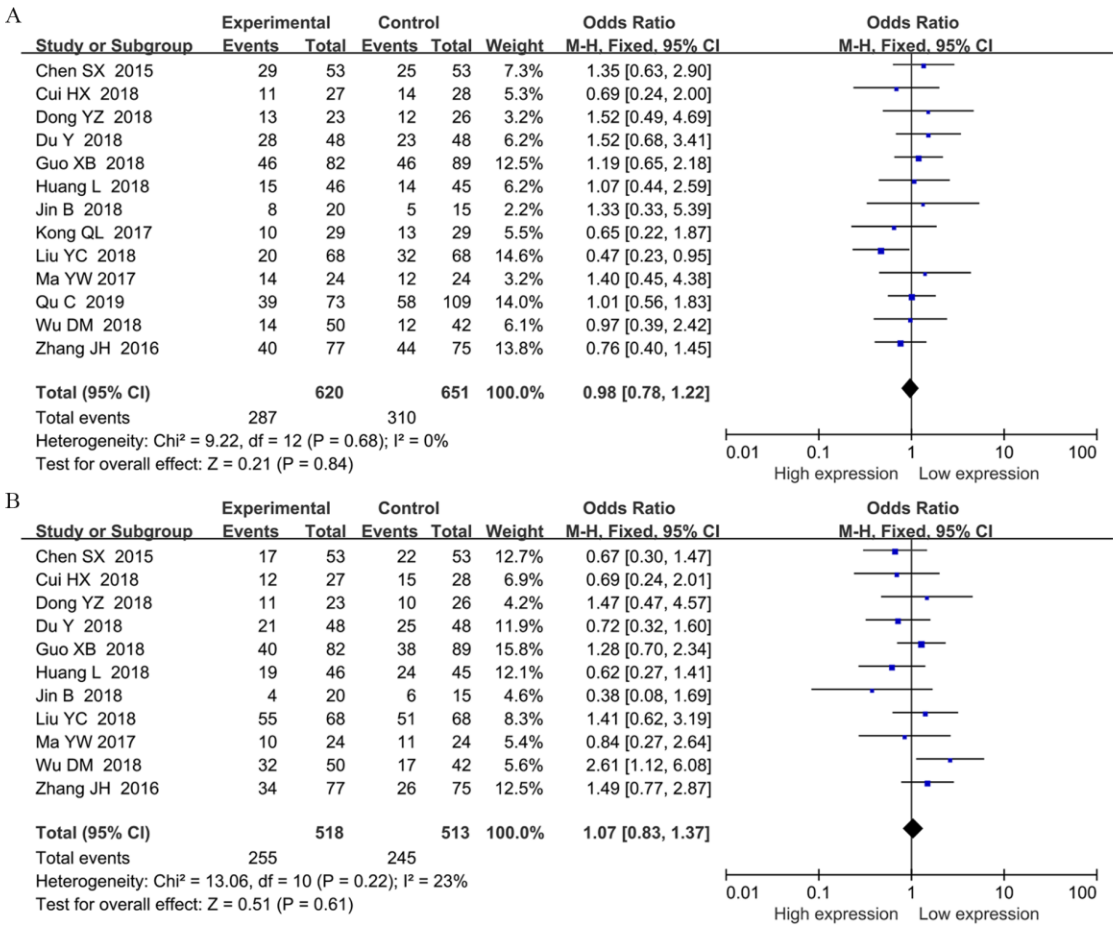

Fig. S4. Forest plot about the relationship between SNHG15 expression and age (older vs. young) (A) and gender (female vs. male) (B).

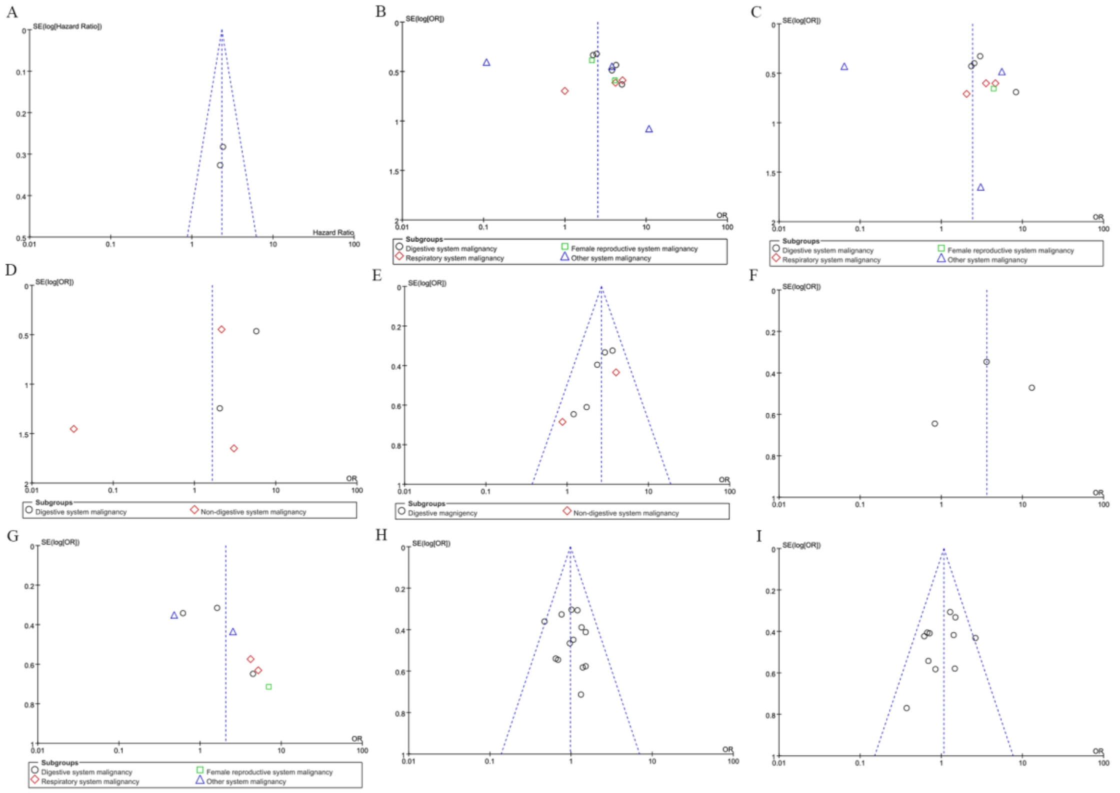

Fig. S5. Funnel plot for the correlation between SNHG15 expression level and different prognosis indicators.DFS (A) .TNM stage (B) .Lymph node metastasis (C) . Distant metastasis (D). Histologic grade (E). depth of invasion (F). Tumor size (G). age (H). Gender (I).

Study or Subgroup	log[Hazard Ratio]	SE	Weight	Hazard Ratio IV, Random, 95% CI
**1.2.1 Lung cancer**				
Cui HX 2018	0.8038	0.3933	8.8%	2.23 [1.03, 4.83]
Dong YZ 2018	0.6152	0.34	10.0%	1.85 [0.95, 3.60]
Jin B 2018	0.4886	0.5451	6.1%	1.63 [0.56, 4.74]
Ma XR 2019	1.1933	0.4507	7.7%	3.30 [1.36, 7.98]
**Subtotal (95% CI)**			**32.6%**	**2.16 [1.44, 3.24]**
Heterogeneity: Tau² = 0.00; Chi² = 1.36, df = 3 (P = 0.71); I² = 0%				
Test for overall effect: Z = 3.73 (P = 0.0002)				
**1.2.2 Hepatocellular carcinoma**				
Zhang JH 2016	0.8096	0.2672	11.8%	2.25 [1.33, 3.79]
**Subtotal (95% CI)**			**11.8%**	**2.25 [1.33, 3.79]**
Heterogeneity: Not applicable				
Test for overall effect: Z = 3.03 (P = 0.002)				
**1.2.3 Thyroid cancer**				
Wu DM 2018	0.6366	0.4516	7.7%	1.89 [0.78, 4.58]
**Subtotal (95% CI)**			**7.7%**	**1.89 [0.78, 4.58]**
Heterogeneity: Not applicable				
Test for overall effect: Z = 1.41 (P = 0.16)				
**1.2.4 Colorectal cancer**				
Huang L 2018	1.0047	0.5101	6.7%	2.73 [1.00, 7.42]
**Subtotal (95% CI)**			**6.7%**	**2.73 [1.00, 7.42]**
Heterogeneity: Not applicable				
Test for overall effect: Z = 1.97 (P = 0.05)				
**1.2.5 Epithelial ovarian cancer**				
Qu C 2019	0.1275	0.031	16.6%	1.14 [1.07, 1.21]
**Subtotal (95% CI)**			**16.6%**	**1.14 [1.07, 1.21]**
Heterogeneity: Not applicable				
Test for overall effect: Z = 4.11 (P < 0.0001)				
**1.2.6 Breast cancer**				
Kong QL 2017	0.6206	0.4305	8.1%	1.86 [0.80, 4.32]
**Subtotal (95% CI)**			**8.1%**	**1.86 [0.80, 4.32]**
Heterogeneity: Not applicable				
Test for overall effect: Z = 1.44 (P = 0.15)				
**1.2.7 Glioma**				
Ma YW 2017	1.3376	1.2329	1.7%	3.81 [0.34, 42.69]
**Subtotal (95% CI)**			**1.7%**	**3.81 [0.34, 42.69]**
Heterogeneity: Not applicable				
Test for overall effect: Z = 1.08 (P = 0.28)				
**1.2.9 Gastric cancer**				
Chen SX 2015	1.0743	0.4127	8.4%	2.93 [1.30, 6.57]
**Subtotal (95% CI)**			**8.4%**	**2.93 [1.30, 6.57]**
Heterogeneity: Not applicable				
Test for overall effect: Z = 2.60 (P = 0.009)				
**1.2.10 Pancreatic cancer**				
Guo XB 2018	1.179	0.5184	6.5%	3.25 [1.18, 8.98]
**Subtotal (95% CI)**			**6.5%**	**3.25 [1.18, 8.98]**
Heterogeneity: Not applicable				
Test for overall effect: Z = 2.27 (P = 0.02)				
**Total (95% CI)**			**100.0%**	**2.07 [1.48, 2.88]**
Heterogeneity: Tau² = 0.17; Chi² = 31.73, df = 11 (P = 0.0008); I² = 65%				
Test for overall effect: Z = 4.27 (P < 0.0001)				
Test for subgroup differences: Chi² = 30.37, df = 8 (P = 0.0002), I² = 73.7%				

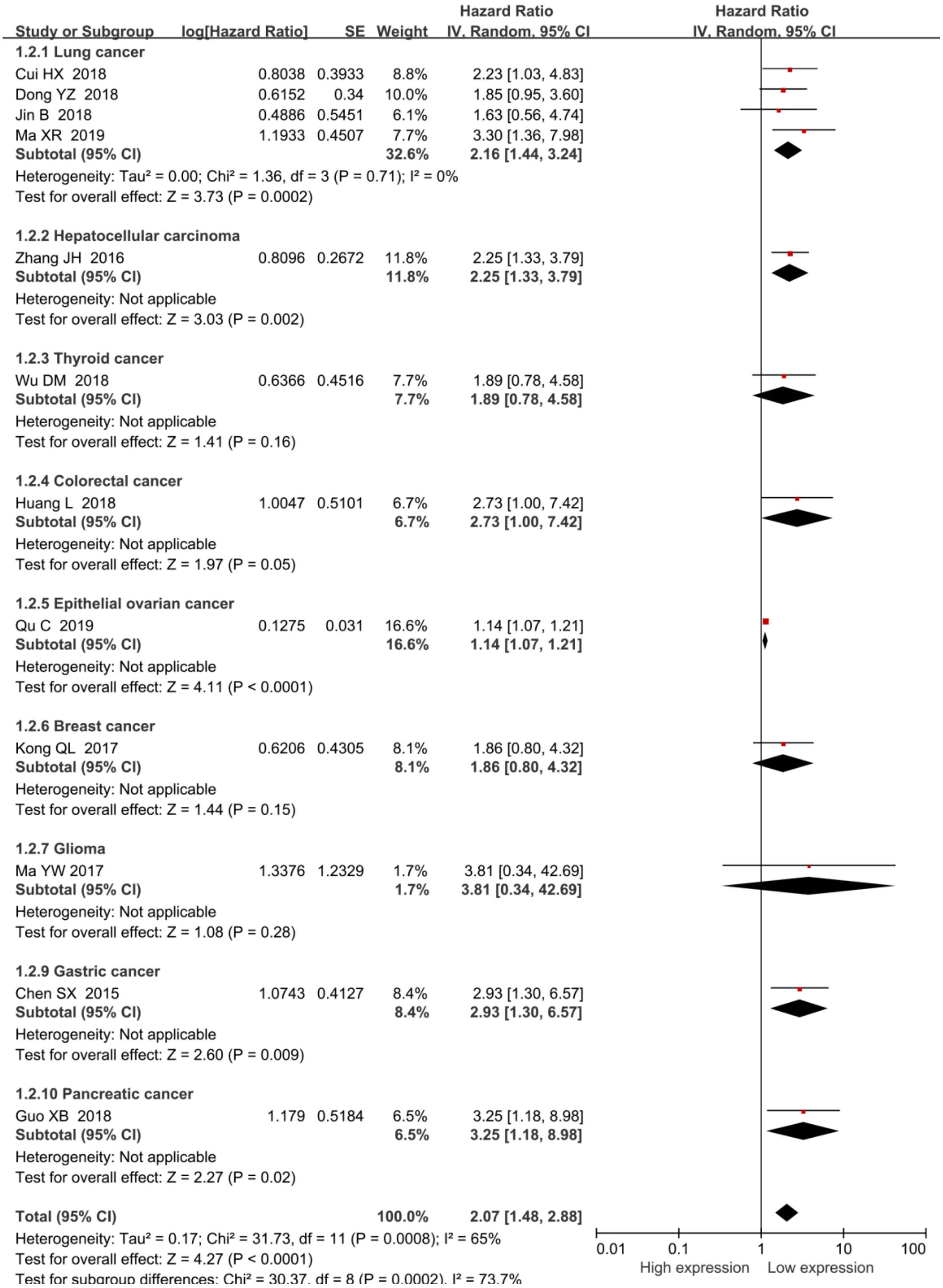

Fig. S6. Forest plot about the relationship between SNHG15 expression and OS in different cancer types.
